# Lead, cadmium, and aluminum in raw bovine milk: Residue level, estimated intake, and fate during artisanal dairy manufacture

**DOI:** 10.5455/javar.2021.h534

**Published:** 2021-09-20

**Authors:** Amr Abd El-Moamen Amer, Hussien Sobhy Abo El-Makarem, Mahmoud Abd-Elsabor El-Maghraby, Sarah Abd-Elmohsen Abou-Alella

**Affiliations:** 1 Department of Food Hygiene and Control, Faculty of Veterinary Medicine, Alexandria University, Alexandria, Egypt; 2 Department of Animal Husbandry and Animal Wealth Development, Faculty of Veterinary Medicine, Alexandria University, Alexandria, Egypt

**Keywords:** Artisanal processing, bovine milk, dairy products, heavy metals, PTWI, THQ

## Abstract

**Objective:**

The goal of this study was to determine the levels of lead (Pb), cadmium (Cd), and aluminum (Al) in raw bovine milk. Milk consumption was used to calculate the estimated weekly intake (EWI), provisional tolerable weekly intake (PTWI), and target hazard quotient (THQ) for individuals. Metal distribution in dairy products and byproducts was studied as a result of artisanal processing.

**Material and Methods:**

Seventy-five raw bovine milk samples were collected from artisanal producers in Alexandria city, West Delta, Egypt, and analyzed using the atomic absorption spectrophotometer. The effect of artisanal dairy processing on metal distribution was studied.

**Results:**

The averages of Pb, Cd, and Al in milk samples were 45.06, 4.77, and 2.93 μg/l, with 13.33% and 1.33% of analyzed samples had Pb and Al levels higher than the permissible limits, respectively. The EWI values of Pb, Cd, and Al were 1.050, 0.111, and 0.068 μg/kg body weight, which contributed to about 4.20%, 1.59%, and 0.97% from the PTWI, respectively. The THQ of three metals was <1, which referred to safe consumption. Metal residues were heavily concentrated in artisanal cheese and yogurt after coagulation and fermentation compared with other dairy products. Accordingly, the maximum average and reduction values of Pb, Cd, and Al were 745.87, 51.99, and 71.58 μg/l and −72.87%, −56.5%, and −40.96% in Damietta cheese; 535.51, 40.11, and 62.43 μg/l and −24.11%, −20.74%, and −22.94%) in Kareish cheese; and 418.42, 31.26, and 50.66 μg/l and 3.02%, 5.90%, and 0.27% in yogurt, respectively.

**Conclusions:**

The results indicated that consumption of raw bovine milk did not pose a risk to Alexandria citizens. Metal concentration increased in artisanal cheese and yogurt because of metal bio-gathering after coagulation and fermentation. Fat separation, churning, and boiling milk might keep metal concentration in dairy products and byproducts at lower levels than milk. Thus, they are suggested to be applied especially in highly contaminated areas.

## Introduction

Milk is a perfect resource of many active biomolecules for people’s health, but it may contain chemical hazards such as heavy metals. The metal residues can get in to the milk due to geological circumstances and environmental releases from several industrial practices nearby the production niches of milk [1]. The main pathway of heavy metals to milk is ingestion by the dairy animal, which constitutes a highly effective biologic barrier against heavy metals from the polluted environment into the milk [2]. The contamination may be directly through pasture and crops used as animal feed or via soil contamination, e.g., cadmium-contaminated phosphate fertilizers. Contamination ways of milk, plants, water, and foods with chemical toxicants, which interfere with the food chain, induce health hazards in humans [3].

The artisanal processing of dairy products was invented in Egypt by the Egyptian Pharaohs and then was developed during Greco-Romans and the Arab Islamic periods [4]. Nowadays, artisanal dairy processing is widely carried out and implemented in huge cities and governorates in lower and upper Egypt. Monitoring of artisanal dairy processing in Egyptian cities should be considered to avoid any pollution by toxic elements. 

Heavy metal concentrations in milk and dairy meals vary dramatically in areas with manufacturing operations and environmentally sanitary districts, allowing them to be utilized as indicators of pollution in the atmosphere and food chain [5].

Heavy metals may be found in dairy products and their byproducts at different levels based on numerous factors, e.g., raw milk contamination, type of processing, entrances, instruments, cooking processes, earthenware, appliances, packaging materials, and water supply applied in dairy plants. Some heavy metals can bind and interact with food components such as protein, minerals, acids, and vitamins [6]. 

The magnitude of heavy metal toxicity can strictly associate with developmental stage, gender, way and time of exposure, weekly intake, biodegradation, absorption level, and elimination mechanisms [7]. In this regard, continuous exposure to a small amount of Pb and Cd even at a low concentration may cause severe health problems such as mental development disorders and irregularities in hematological function via limiting the formation of hemoglobin and shortening the lifespan of red blood cells [8]. Additionally, Pb and Cd can possess potent carcinogenic activity against vital tissues of the human body, especially cardiovascular, nervous, renal, and skeletal systems [9]. In children, the toxic level of Pb can cause severe dementia, headaches, convulsions, memorial disturbances, behavioral disorders, anxiety, immobilization, and unconsciousness. Death can occur due to neural damage, edema, and hematological changes [10]. Cd in a minor concentration in food of grazing animals, particularly plants and grasses, can lead to bioaccumulation of this toxic metal in their body tissues. Cd may cause several metabolic syndromes in humans, such as renal damage, bone fractures, apoptosis, oxidative stress, and cancer [11]. Furthermore, aluminum is considered a neurotoxic metal and acts as a cofactor in Alzheimer’s disease in humans. Aluminum toxicity can cause neurodegenerative disorders and osteomalacia [12].

Toxic metal residues in raw milk have been investigated in highly industrialized communities such as South Korea [13], Pakistan [14], Croatia [15], Brazil [16], Poland [17], and Slovakia [18]. Although the amount of metals in raw bovine milk is small, their concentration can be meaningfully modified toward either elevation or reduction by using various artisanal processing techniques such as fat separation, churning, boiling off, fermentation, and cheese processing. Other factors may affect the metal concentration, e.g., temperature, pH value, heat treatment, and some additives such as organic acids, salt, and other ions [19].

Abd-El Aal et al. [20] in El Dakahlia governorate, Meshref et al. [21] in Beni-Suef governorate, Eleboudy et al. [22] in Alexandria governorate, and Saleh et al. [23] in Behera governorate investigated heavy metal residues in various types of milk (raw, sterilized, and processed) and some dairy products. However, research evaluating the goal hazard quotient for safe milk intake by Egyptian consumers is lacking. In addition, this study is unique as it closely covered the residual level of examined metals throughout nearby all experimental dairy processing approaches, which are usually applied to convert milk into artisanal dairy products and their byproducts. Hence, there is a substantial necessity to choose the appropriate artisanal milk processing that can minimalize metal concentration, especially in highly contaminated areas.

The present research was planned to monitor the levels and frequency of detecting Pb, Cd, and Al residues in raw bovine milk of artisanal producers at Alexandria, west delta, Egypt, and match them with the maximum permissible limits (PLs). Additionally, we assess the health risk indices based on measuring the estimated weekly intake (EWI), the provisional tolerable weekly intake (PTWI), and the target hazard quotient (THQ) of those metals for adults persons through raw bovine milk consumption. Aside from the aforementioned goals, the study looked into the impact of artisanal milk processing on the distribution of heavy metals in dairy products and byproducts, as well as whether fat separation, churning, boiling off, cheese manufacturing, and fermentation processes can lead to an increase or decrease in residual levels.

## Materials and Methods

### Description of the sampling sites

**Figure 1.**
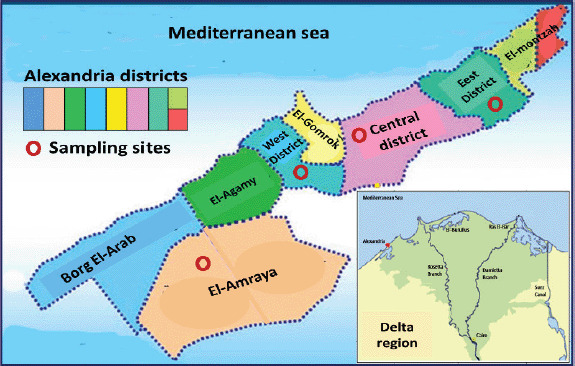
Schematic map of sampling sites in Alexandria city, West Delta, Egypt, including four districts: El-Amraya, West, Central, and East districts.

The area of the Delta is positioned at the North of Egypt, and it resembles an umbrella (Fig. 1). It is divided into four main portions: West, East, North, and South of Delta, where Alexandria city is in the West Delta region. This city has a damp atmosphere with a yearly normal temperature range of 25°C–32°C in the summer and 10°C–16°C in wintry weather with heavy rains. Alexandria is a coastal city as its great portion is localized nearby the Mediterranean Sea. It consists of eight chief districts (Borg El-Arab, El-Amraya, El-Agamy, West, Central, and East districts, El-Gomrok, and El-Montazah) (Fig. 1). The artisanal dairy products are distributed at a wide level in all mentioned districts. Concerning the development of the industry, some districts like Borg El-Arab and El-Amraya have higher industrial activities. Others have moderate to low activities with various levels of toxic metal contamination (e.g., Pb, Cd, and Al) in the areas surrounding milk and dairy products. Large levels of heavy metals have been dispersed into the atmosphere, soil, drinking water, and utensils, which are the principal sources of contamination for foods, since the industrial revolution began many years ago in Alexandria, posing serious public health concerns to consumers.

Four sampling sites were carefully chosen in Alexandria city (Fig. 1): El-Amraya, West, Central, and East districts. These areas were augmented by artisanal dairy production and distribution. Furthermore, many small dairy farms, retail stores, and supermarkets were localized in these areas where artisanal milk and dairy products can be sold to local citizens. Consequently, raw bovine milk samples (75 in number) were gathered randomly from artisanal producers in four districts at Alexandria city, West Delta, Egypt. The samples (250 ml) were obtained as sold to the public in screw-topped bottles or polyethylene containers and transported within an hour in an icebox at 4°C ± 1°C to the laboratory and preserved at −20°C up to the assessment.

### Assessment of heavy metal level

The collected samples were examined to determine the lead, cadmium, and aluminum level based on wet weight (μg/l). Many reagents and glassware used in the experiment were obtained from Sigma-Aldrich Ltd., Egypt.

### Digestion technique

Two grams of each sample was digested by 10 ml of digestion mix (60 ml, nitric acid 69% added to 40 ml, perchloric acid 70%; PubChem, Rockville Pike, MD) in a screw-capped tube [24]. The tubes were firmly locked, and the insides were intensely agitated then kept at an ambient temperature nightlong. Moreover, the tubes were allowed to be boiled for 4 h in a water bath (SW22, AC/DC input 230 V AC; Sigma-Aldrich Ltd., Egypt) starting from 60°C till reaching 110°C for complete digestion of the samples. The digestion tubes were tightly stirred at 30 min interims throughout the process of heating. All tubes were kept for cooling and reaching the surrounding temperature, then watered down using 1 ml, demineralized water 30% (Sigma-Aldrich, Egypt) and heated again in the water bath at 70°C to confirm extensive digestion. At this step, all organic matrices have been damaged. 25 ml water was added to each tube, and the sample was clarified with filter paper No. 42 (Whatman, 1442-042, 0.40 psi wet burst; Sigma-Aldrich Ltd., Egypt). The filtrates were gathered in glass test tubes covered by polyethylene tape and reserved at surrounding ambient temperature till tested for the heavy element concentrations.

### Preparation of blank and standard solutions

Helpful processes for different assessments were built on those recommended in the operative instruction manual of the atomic absorption spectrophotometer (ZA3000 series- Hitachi, Ltd., Chiyoda-ku, Tokyo, Japan). However, blank and standard solutions were made the same way as performed for moist digestion and by applying the same chemicals reported by Shibamoto and Bjeldanes [25]. The blank solution consisted of nitric acid (10 parts) and hydrogen peroxide (H_2_O_2_) (1 part), then was liquified with deionized water (25 parts) and purified by filtration. The blank was applied to verify the metal contamination of the chemicals, and its level was excluded from the end results. Furthermore, standard solutions containing clear qualified metal standards at varied concentrations were created by combining 10 parts nitric acid and 1 part H_2_O_2_, followed by 25 parts demineralized water. Glass and plastic equipment were cleaned and preserved overnight in 10% (v/v) nitric acid, then dipped in deionized water and dried.

### Samples analysis

The digest, blanks, and standard solutions were extracted by flame atomic absorption spectrophotometer (Varian, AA240 FS, Pty. Ltd., Victoria, Australia) using an adjunct initiator (HG 3000, Pty. Ltd., Victoria, Australia). The device has an automatic sampler digital absorbance and concentration readout able to operate under the following circumstances advised by the instrumental manual:

**Table:** 

Heavy metal condition	Lead	Cadmium	Aluminum
Lamp wavelength (nm)	283.3	228.8	275.6
Lamp current (m/amp)	10	4	12
Fuel flow rate	1.4	1.2	1.4
Used gas	Argon	Argon	A–AC^*^
Measurement time (seconds)	4.0	4.0	4.0
Detection limit (ppb)	8–40	0.2–0.8	0.5–1.0

N_2_O/A/AC^ *^ = Nitrous oxide/air/acetylene.

### EWI of heavy metals

We have quantified EWI as described previously by Kabir et al. [26] in Equation (1).

1 $$\mathrm{Estimated}\;\mathrm{daily}\;\mathrm{int}\mathrm{ake}\;(\mathrm{EDI})=\frac{C_{\mathrm{metal}}\times W_{\mathrm{food}}}{\mathrm{BW}}=\mu g/\mathrm{kgbw}/\mathrm{day}$$

EWI = EDI × 7

where *C*_metal_ (mg/kg) is the average metal level in the tested milk sample, *W*_food_ represents the mean food consumption/day, and (BW) signifies the body weight. The average intake of milk each day per mature individual (60 kg BW) was evaluated to be 200 ml [27]. The EDI of metal (mg/day) from milk was calculated and multiplied by 7 to obtain the EWI.

### Target hazard quotient

The THQ associated with the native populations via drinking contaminated milk by heavy metals was evaluated using Equation (2) according to Kabir et al. [26] built on the reference oral dose (RFD_o_) for each metal. THQ < 1 referred to the absence of toxicity and expected safety for the exposed population [7].

RFD_o_ is the evaluation of daily oral exposure for the population, which has an unharmful effect on humans throughout life [28]. Oral reference doses were based on 3.5E-03, 1.00E-03, and 4.00E-04 mg kg^−1^day^−1^ for Pb, Cd, and Al, respectively [29,30].

2 $$\mathrm{THQ}=\frac{\mathrm{EDI}(\mathrm{mg}/\mathrm{kg}/\mathrm{day}}{{\mathrm{RFD}}_{o}(\mathrm{mg}/\mathrm{kg}/\mathrm{day})}$$

### Fate of metal residues in experimental artisanal dairy products

Twenty liters of raw milk were acquired from the dairy farm of the Faculty of Agriculture, Alexandria University. According to Abou-Donia, milk was divided into five equal parts to manufacture cream, butter, Kareish cheese, Damietta cheese, and yogurt [4]. Milk, dairy products, and their byproducts were analyzed to determine Pb, Cd, and Al contents (μg/l) based on wet weight using flame atomic absorption spectrophotometer as mentioned before.

### Data analysis

The data were described using the median, 25th, and 75th percentiles, as well as the mean and standard deviation, due to the non-normality of the data (several zero values). All calculations were implemented based on Statistical Package for the Social Sciences (SPSS) statistical package (IBM SPSS Statistics for Windows, IBM Corp., Armonk, NY).

## Results and Discussion

### Residue level of lead in raw bovine milk samples

The concentrations and frequency of detection for heavy metals (Pb, Cd, and Al) in raw bovine milk samples of artisanal producers at Alexandria city, West Delta, Egypt, are reported in Table 1. All three metals were detected at varying concentrations in the order of Pb > Cd > Al in all contaminated samples. In this regard, the Pb level in tested milk samples varied between 0 and 630 μg/l with a mean concentration (standard deviation, SD) of 45.06 (128.9) μg/l. Only 13.33% (N:10) of the samples contained Pd at a concentration exceeding the PL (20 μg/l) established by the Egyptian Organization for Standardization and quality 7136/2010 [31] and European Union [32]. However, a contaminated sample contained approximately 17 times higher concentrations of Pb than the PL. Accordingly, the least and highest levels of Pb (μg/l) in contaminated samples were 130 and 630 with a mean concentration (SD) of 338.0 (156.2) μg/l, while the levels were 242 and 442 with a median value of 300.0 μg/l, respectively. Also, all positive samples had Pb levels above the Chinese maximum residue limit (50 μg/l) stated by the China Food and Drug Administration and National Health Commission [33]. The mean value of Pb in all examined samples reported herein is relatively higher than those obtained for raw milk by Bilandžić et al. [15] in Croatia (11.4 μg/l), Singh et al. [34] in India (6.10 μg/l), Sujka et al. [17] in Poland (13 μg/l), and Castro-González et al. [35] in Mexico (24 μg/l). Furthermore, this result is comparatively greater than those shown by Oliveira et al. [16], who found that the Pb level in milk samples obtained from Brazilian manufacturers ranged from 2.12 to 37.36 μg/l. On the other hand, this result is relatively lower than the values of Kazi et al. [36] for Pakistani raw milk (47.6 μg/l), Ahmad et al. [37] for Bangladeshi raw milk (170 μg/l), Akele et al. [38] for Ethiopian raw milk (153 μg/l), and Pšenková et al. [18] for Slovakian raw milk (100 μg/l). In Egypt, the average values of Pb in raw cow milk investigated by Meshref et al. [21], El-Bassiony et al. [39], Saad [40], Khalil [41], and Saleh et al. [23] were higher than our study (214, 201, 130, 319, and 110 μg/l, respectively). On the contrary, Malhat et al. [42] found a lower Pb value (1.85 μg/l) for cow milk.

**Table 1.** Concentration (μg/l) and frequency of the detection of lead, cadmium, and aluminum residues in samples of raw milk collected from artisanal producers at West Delta, Egypt.

Metals	All samples				EWI μg/kg BW/week	Contribution to PTWI%	THQ				Contaminated samples		
	*N*	Min–max	Mean (SD)	Median (Q1–Q3)				*N* (%)	*N* (%) > PL	PL μg/l	Min–max	Mean (SD)	Median (Q1–Q3)
Lead	75	0–630	45.06 (128.9)	0 (0–0)	1.051	4.20	0.04	10 (13.33)	10 (13.33)	20	130–630	338.0 (156.2)	300 (242–442)
Cadmium	75	0–80	4.77 (14.79)	0 (0–0)	0.111	1.59	0.015	9 (12.00)	ns	ns	10–80	39.77 (21.41)	40 (23.5–55)
Aluminum	75	0–50	2.93 (9.69)	0 (0–0)	0.068	0.97	0.22	7 (9.33)	1 (1.33)	50	20–50	31.42 (10.69)	30 (20–40)

*N*: number of examined samples; Min–max: minimum–maximum; Q1: 25th percentile, Q3: 75th percentile; PL: permissible limit as per the EOSQ (2010) No 7136/2010: lead: 20 μg/l, IPCS (2009): aluminum: 50 μg/l, ns: not set, SD: standard deviation; EWI: estimated weekly intake; PTWI: provisional tolerable weekly intake; THQ: target hazard quotient.

Elevated concentration of Pb in contaminated milk samples might be due to contaminated irrigation systems, extensive use of pesticides, accumulation along roads and motorways, climatic factors, and ecological sources (atmospheric deposition, waste removal, and exhausted vehicle) [43]. 

Regarding modern industrialization of the area under investigation, herein was Alexandria city at West Delta of Egypt; hence, PB is an airborne pollutant and can be transported to the air at various levels depending on industrial activities of a region or a city. As a result, an expectation of rising Pb levels in the raw milk environment and contamination may have arisen from drinking water, livestock feeding, dust, soil, paints, enameled soil cookware, metal bowls, milk pipelines, cosmetics, insecticides, batteries, gasoline, traffic flow, mining factories, and printing houses where Pb is used. Lead can persist in the environment and be transferred to milk then, which may cause severe toxic health impacts after consumption [44]. Furthermore, the other thing of various Pb residues in milk compared to other studies is the status of milk payment system where this study focused on an artisanal producer who has his/her small scale farm and either can use the milk or retail it to the public with lacking awareness about the handling of milk during milking, processing, and transportation.

### Residue level of cadmium in raw bovine milk samples

In the current study, 9 (12%) samples had detectable Cd levels, and their concentration in all tested samples ranged between 0 and 80 with a mean concentration (SD) of 4.77 (14.79) μg/l. Unfortunately, there is no PL available for Cd in raw milk. The frequency of Cd detection among contaminated samples was in the range of 10 and 80, with a mean concentration (SD) of 39.77 (21.41) μg/l, while the range for median (40 μg/l) was 23.5–55 μg/l.

The obtained value is considerably lower than earlier findings in Egypt reported by Al-Ashamawy et al. [27], Abd-El Aal et al. [20], Malhat et al. [42], Meshref et al. [21], El-Ansary [45], Khalil [41], and Saleh et al. [23] who investigated that the means of Cd residue in raw milk were 52, 416, 288, 51, 31.5, 306, and 30 μg/l, respectively. Globally, this result is relatively lesser than those investigated by Parween et al. [46] in Pakistan (53 μg/l), Singh et al. [34] in India (11.17 μg/l), Sujka et al. [17] in Poland (5.8–6.7 μg/l), and Zhou et al. [47] in China (44.2 μg/l) for raw milk. However, it is comparable with the means of 1.8, 2, and 1 μg/l obtained by Hafez and Kishk [48] in Egypt, Rahimi [8] in Iran, and Ismail et al. [14] in Pakistan, for raw milk, respectively. This finding nearly agrees with Pšenková et al. [18], who reported that the average level of Cd in raw milk was 4 μg/l. On the other hand, our result is above the values reported by Castro-González et al. [35] (2 μg/l) for raw milk.

It is presumed that the possible source of environmental pollution of milk with Cd, especially in highly industrialized cities like Alexandria, may be through galvanized pipes and effluent from electroplating workers and geological deposits and steel industry, fossil fuels, and traffic activities [49]. Significantly, the Cd level in milk from Alexandria city may originate from soil contamination with Cd and increasing industrial activities from which Cd can migrate into milk. It is essential to consider that Cd is highly cumulative and has a prolonged shelf life-extending for years; thus, consuming foods contaminated with Cd even in small quantities for a long time can cause renal and respiratory disorders [50].

### Residue level of aluminum in raw bovine milk samples

The findings in Table 1 reveal that the average concentration (SD) of Al in the tested milk samples was 2.93 (9.69) μg/l with a range of 0 and 50 μg/l. Only a single (1.33%) sample exceeded the permissible content (50 μ/l) of Al set by the International Program on Chemical Safety [51]. The range of Al residue among contaminated samples was 20–50 μg/l, with an average level (SD) of 31.42 (10.69) μg/l, while the interquartile range was 20–40 μg/l, with a median value of 30 μg/l. This indicates low contamination levels of raw milk by Al residue in the area under investigation (Alexandria). Other Egyptian authors reported much higher levels of Al, e.g., El-Mossalami and Noseir [52]: 840 μg/l, Al-Ashamawy [27]: 81 μg/l, and Abd-El Aal et al. [20]: 501 μg/l.

Aluminum residues in raw milk can be caused by the extensive utilization of Al containers along the milk chain (production, collection, storage, and carriage of milk from dairy plants to retails and supermarkets). In Egypt, including the West Delta region, milk in marketplaces is frequently obtained from artisanal producers who usually use low-quality tools for boiling milk, such as Al vats with inappropriate design to prolong its quality. They may store it in milk coolers for the next day and then added to the freshly collected milk. Therefore, this creates accidental leaching of Al from vats to milk, which is affected by the condition of Al containers and pH value [53]. In addition, water may be added to milk as a source of impurity, containing Al residues [54].

### EWI of heavy metals

An EWI has been matched with the PTWI to evaluate the possible health risk related to toxic metal contamination of milk. The Joint FAO/WHO Expert Committee on Food Additives (FAO/WHO) [55] established PTWI of heavy metals to emphasize the cumulative nature of these toxic metals. 

The data in Table 1 explained that the trends of EWIs and their contribution to PTWI for examined metals in raw bovine milk were ordered as Pb > Cd > Al. Consequently, the EWI values of Pb, Cd, and Al for a mature human (60 kg BW) feeding 200 ml raw bovine milk each day as established by FAO [56] and Nutrition Institute Cairo [57], were 1.051, 0.111, and 0.0680 μg/kg BW/week, correspondingly. This donates about 4.20%, 1.59%, and 0.97% of the PTWI levels endorsed by the Joint FAO/WHO Expert Committee on Food Additives [55,56] for Pb and Cd to be 25 and 7 μg/kg BW/week, respectively, while PTWT of Al set by WHO [58] to be 7 μg/kg BW/week. The EWI in this result is smaller than those described by Al-Ashamawy [27], who calculated the EWI of Al for a mature individual in Dakahlia governorate, Egypt, to be 196 μg/kg BW/week, which contributed about 3.0% of the provisional tolerable daily intake. Furthermore, this finding is relatively lower than EWI of Pb and Cd quantified by Meshref et al. [21] with values of 8.89 and 2.31 μg/kg BW/week, respectively, for adults in Beni-Suef governorate, Upper Egypt. Similarly, Khalil [41] found that the EWI values of Pb and Cd were 9.8 and 8.4 μg/kg BW/week, respectively, in Aswan governorate, Upper Egypt. This study showed that the EWI of Pb was higher than Cd, and this result is in line with that reported by Ghafari and Sobhanardakani [59]. 

The EWI was quantified for Pb, Cd, and Al in milk, which only constituted a part of the contamination over everyday consumption of the whole diet. The dietary exposure of investigated metals for native Egyptians in diverse parts of Egypt, which may have minimal industrialized activities such as Upper Egypt or vast industrial capacities such as the West Delta including Alexandria, may achieve inaccurate rates. So, the investigation of the entire regimen for the people of Alexandria is required for evaluating the actual toxic risk. Finally, withdrawing the PTWI for examined metals by FAO/WHO experts [55] has left it essential to search for other approaches to assess the risk related to food consumption with substantial levels of these and other toxic metals.

### THQ of heavy metals

The results of THQ of three metals via milk consumption are described in Table 1. All values were less than one (safe limits), declaring that consumption of raw milk does not constitute a possible wellbeing risk to the citizens in Alexandria city, West Delta, Egypt. Accordingly, the THQ values of Pb, Cd, and Al via raw milk consumption were 0.04, 0.111, and 0.97, respectively. The THQ is a proportion of a specified contaminant dosage to a RFD_o_ for that substance [7]. In this context, the RFD_o_ for Pb, Cd, and Al were 3.5 E-03, 1.00E-03, and 4.00E-04 mg kg^−1^day^−1^, respectively. The THQ has been proved a valuable index for assessing risk linked to the ingestion of metal-containing food. Nevertheless, they may be exposed to metal contamination via dust inhalation, direct contact, and any contaminated foods and drinking water consumption. This finding is in line with that reported by Meshref et al. [21]: Beni-Suef governorate; Upper Egypt, Khalil [41]: Aswan governorate; Upper Egypt, and Sobhanardakani [60]: Hamadan city, western Iran, for THQ of Pb and Cd via raw milk consumption to be less than one. 

The obtained findings in this study indicate that the residents in Alexandria, West Delta of Egypt, will not be subjected to a possible wellbeing threat from drinking raw milk. Nevertheless, additional potential sources of metal exposure should be considered, such as dust inhalation, direct contact, and consumption of any contaminated foods and drinking water, which were not involved in our study. So other monitoring policies are needed to counteract the metal levels in the surrounding environment of milk to ensure maximal human protection against heavy metal risks.

### The fate of Pb, Cd, and Al residues in experimental artisanal dairy products

The results in Table 2 describes the fate of Pb, Cd, and Al in raw bovine milk, experimentally manufactured artisanal dairy products (cream, butter, ghee, Kareish cheese, Damietta cheese and its additives (rennet and salt), and yogurt, in addition to their byproducts (skimmed milk, buttermilk, murta (curd), and whey of manufactured cheeses). The analysis applies different artisanal processing treatments such as fat separation, churning, boiling off, cheese making, and fermentation. The fate of examined metals was monitored by measuring their mean concentrations and corresponding reduction % after processing. Accordingly, there was a great variation in the values among dairy products when compared with original milk. The mean concentration levels (μg /l): reduction values (%) of Pb amongst dairy products and byproducts compared with raw milk (431.46: 0) were in the following order: Damietta cheese (745.87: −72.87%) > Kareish cheese (535.51: −24.11%) > yogurt (418.42:3.02%) > cream (297.62:31.02%) > murta (257.88:40.23%) > buttermilk (245.10:43.19%) > Damietta cheese whey (221.08:48.76%) > Kareish cheese whey (218.38:49.38%) > butter (211.74:50.92%) > skimmed milk (173.66:59.75%) > ghee (116.26:73.05%). 

Regarding the fate of Cd after milk processing, the mean concentrations (μg/l): reduction percentages (%) of Cd in dairy products and byproducts incomparable of raw milk (33.22:0) were in the following direction: Damietta cheese (51.99: −56.50%) > Kareish cheese (40.11: −20.74%) > yogurt (31.26:5.90%) > cream (22.80:31.36%) > buttermilk (15.62:52.98%) > Damietta cheese whey (15.45:53.49%) > butter (14.38:56.71%) > Kareish cheese whey (13.08:60.62%) > murta (11.48:65.44%) > skimmed milk (10.04:69.77%) > ghee (8.46:74.53%). 

**Table 2.** Fate of lead, cadmium, and aluminum residues (μg /kg) in laboratory manufactured artisanal dairy products.

Manufacturing process	Concentrations of heavy metals (μg /kg) (Mean ± SD)					
	Lead	*R*%^a^	Cadmium	*R*%^a^	Aluminum	*R*%*
Raw cow milk	431.46 ± 120.12		33.22 ± 5.89		50.78 ± 2.26	
Fat separation						
Cream	297.62 ± 18.68	31.02	22.80 ± 5.08	31.36	39.81 ± 2.03	21.60
Skim milk	173.66 ± 15.96	59.75	10.04 ± 1.66	69.77	20.13 ± 1.58	60.35
Churning						
Butter	211.74 ± 18.47	50.92	14.38 ± 1.48	56.71	26.29 ± 1.03	48.23
Butter milk	245.10 ± 35.30	43.19	15.62 ± 2.42	52.98	29.77 ± 1.07	41.37
Boiling off						
Ghee	116.26 ± 13.31	73.05	8.46 ± 1.52	74.53	16.52 ± 1.67	67.46
Murta (curd)	257.88 ± 25.74	40.23	11.48 ± 1.43	65.44	20.54 ± 1.56	59.55
Cheese manufacturing						
Kareish cheese	535.51 ± 85.34	-24.11	40.11 ± 1.28	-20.74	62.43 ± 1.46	-22.94
Whey Kareish cheese	218.38 ± 19.87	49.38	13.08 ± 1.21	60.62	25.40 ± 1.84	49.98
Damietta cheese	745.87 ± 70.01	-72.87	51.99 ± 2.09	-56.50	71.58 ± 1.61	-40.96
Rennet	210.38 ± 17.47		28.30 ± 1.02		19.48 ± 0.72	
Salt	270.36 ± 21.67		22.45 ± 1.39		16.52 ± 0.81	
Whey Damietta cheese	221.08 ± 25.23	48.76	15.45 ± 1.46	53.49	17.37 ± 2.66	65.79
Fermentation						
Yogurt	418.42 ± 122.11	3.02	31.26 ± 1.42	5.90	50.66 ± 2.24	0.27

*R*%^a^ = reduction percent.

Concerning the Al content, the mean values (μg /l): reduction levels (%) of Al in dairy products and byproducts matched with raw milk (50.78:0) were in the following order: Damietta cheese (71.58:−40.96%) > Kareish cheese (62.43:−22.94%) > yogurt (50.66:0.27%) > cream (39.81:21.60%) > buttermilk (29.77:41.37%) > butter (26.29:48.23%) > Kareish cheese whey (25.40:49.98%) > murta (20.45:59.55%) > skimmed milk (20.13:60.35%) > Damietta cheese whey (17.37:65.79%) > ghee (16.52:67.46%). The mean concentrations of toxic metals in rennet and salt (additives of Damietta cheese) were 210.38 and 270.36 μg /l for Pb, 28.30 and 22.45 μg /l for Cd, and 19.48 and 16.52 μg /l for Al, respectively. 

According to the previously mentioned results, the levels of examined metals were considerably higher in Kareish cheese, Damietta cheese, and yogurt than in other dairy products and their byproducts. This may be due to increasing protein content as metals have binding ability to functional groups of casein, such as sulphydryl, carboxyl, amino and peptide groups, and reducing the moisture content during cheese making [61]. Also, the levels of metals in cheese can depend on the manufacturing process; hence Damietta cheese in our study contained a higher value of examined metals than Kareish cheese. This is because of adding some additives such as rennet and salt during Damietta cheese making, which may contain extra metals and hence may cause an increase of the metal level in the final cheese [62]. Briffa et al. [63] reported that Pb has a high affinity to bind with the sulfhydryl group of the protein (casein).

These findings agree with those obtained by Amer [64], who investigated that the levels of Pb, Cd, and Al in cheese (Kareish and Damietta) were higher than those in cream and butter. In addition, Castro-González et al. [61] found that the concentration of Pb during cheese manufacture increased about five times more than the original Pb-contaminated milk. Also, Mehennaoui et al. [65] reported that most Cd in milk was related to casein fractions. Nearly 60% of milk Cd moved to the curd while 14% was transferred to the whey. In Poland, Sujka et al. [17] found that yogurt and cheese samples were highly contaminated with Pb than original milk (13 μg/kg) in the ranges from 99 to 156 μg/kg for yogurt, and 340 to 380 μg/kg for cottage cheeses.

Practically, fermentation has little or no effect on heavy metal content. Accordingly, the levels of Cd and Al in manufactured yogurt were nearly similar to milk except for Pb value in yogurt was equivalent to or greater than in milk. This finding is in line with that reported by Khan et al. [13], who noted that the Pb levels in yogurt (4.21–24.50) > milk (3.35) ng/g. The moderate level of metals in cheese whey indicates that most of them remain bound to cheesy matrices, with a bit of fraction escape with the whey.

The food’s nutritional quality plays a crucial role in Al leaching from processing containers. Therefore, the manufacture of cheese, highly acidic products (yogurt) can enhance Al leaching into the food from utensils. A comparable investigation has also been shown by Semwal et al. [66]. In contrast, El-Barbary and Hamouda [67] showed that the Al level decreased during cheese processing from 561 μg/kg in raw milk to 401 μg/kg in fresh cheese. 

In contrast, boiling off was associated with a relatively higher reduction% in metal contents than other manufacturing processes; thus, ghee contained the lowest amount of all examined metals, while murta had tiny amounts. The variation in metal levels after boiling off milk may be attributed to the creation of compounds between the whey proteins and the metal or to the desegregation of the metal-bound to casein particles [68].

Low metal concentration in butter might be attributed to the remarkable ability of metals to combine with the protein fraction so, buttermilk contained the greatest concentration of metals which could be increased by the advancement of time. Our result agrees with those obtained by Vahedi et al. [69], who found that the Pb level transferred into buttermilk (862 μg/kg) was higher than butter (849 μg/kg) and cream (839 μg/kg). The authors confirmed that Pb and Cd had high tendencies toward the serum phase (buttermilk) during butter manufacturing. Regarding the fat separation process, it is observed that the level of metals in cream was greater than skimmed milk. The majority of the Pb, Cd, and Al were recovered in the cream during separation, with only a small quantity escaping into the milk. This means that metals have an exceptional ability to attach to protein and membrane lipoproteins in cream fat globules.

Moreover, other factors enhancing metal accumulation such as utensils, additives, and developed acidity during cheese making will increase corrosion of Al from vats. Regarding artisanal dairy processing, contamination by heavy metals may happen at numerous phases throughout processes, e.g., from dairy entrances, dairy plants, catering processes, utensils, packaging materials, and water applied in dairy manufacturing. Finally, our study is unique as it considered all processing approaches used for artisanal dairy products in the West Delta of Egypt. It is essential to choose the ideal processing way to decrease the metal level in dairy products to ensure food safety and quality.

## Conclusion

In summary, artisanal raw bovine milk has a reasonably low frequency of contamination with Pb, Cd, and Al. However, positive samples for Pb contained extremely high levels relative to the PL. Intake and health risk indices indicate that milk intake does not cause a health hazard to local inhabitants of Alexandria. When milk was processed into artisanal dairy products, heavy metals are likely to increase upon cheese and yogurt production, which could result from metal bio-gathering after coagulation and fermentation. Other processes (fat separation, churning, and boiling off milk) can reduce metal concentration in dairy products and byproducts at lower levels than raw milk. Consequently, unique inspection should be maintained on metal residues in milk. When they are found at a level above the PL, it may enhance milk processing into dairy products with efficient metal monitoring in the processed dairy products. Storage and processing in metal-containing utensils raise some heavy metals, mainly, Al in the product. Preventive strategies involving all surrounding circumstances of milk (proper handling, utensils, and appropriate processing) should be directed for areas at high risk of heavy chemical exposure. The actual training of the artisanal producers to increase attentiveness of heavy metals and their hazards should be observed. Future investigations will be necessarily applied to assess, bio-monitor the metal level in other areas of Egypt within the safety and PLs. Finally, periodical surveillance should be suggested, especially in highly industrialized areas, to ensure consumers’ safe dietary intake of milk.

## List of Abbreviations

Al, Aluminum; BW, Body weight; Cd, Cadmium; EDI, Estimated daily intake; EWI, Estimated weekly intake; Pb, Lead; PLs, Permissible limits; PTWI, Provisional tolerable weekly intake; RFD_o_, Reference oral dose; SD, Standard deviation; THQ, Target hazard quotient.
